# Does the drug sensitivity of malaria parasites depend on their virulence?

**DOI:** 10.1186/1475-2875-7-257

**Published:** 2008-12-16

**Authors:** Petra Schneider, Brian HK Chan, Sarah E Reece, Andrew F Read

**Affiliations:** 1 Institutes of Evolution, Immunology and Infection Research, University of Edinburgh, EH9 3JT, UK; 2 Centre for Immunity, Infection and Evolution Ashworth Laboratories, School of Biological Sciences, University of Edinburgh, EH9 3JT, UK; 3 Center for Infectious Disease Dynamics, Department of Biology, Mueller Laboratory, the Pennsylvania State University, University Park, PA 16802, USA

## Abstract

**Background:**

Chemotherapy can prompt the evolution of classical drug resistance, but selection can also favour other parasite traits that confer a survival advantage in the presence of drugs. The experiments reported here test the hypothesis that sub-optimal drug treatment of malaria parasites might generate survival and transmission advantages for virulent parasites.

**Methods:**

Two *Plasmodium chabaudi *lines, one derived from the other by serial passage, were used to establish avirulent and virulent infections in mice. After five days, infections were treated with various doses of pyrimethamine administered over 1 or 4 days. Virulence measures (weight and anaemia), parasite and gametocyte dynamics were followed until day 21.

**Results:**

All treatment regimes reduced parasite and gametocyte densities, but infections with the virulent line always produced more parasites and more gametocytes than infections with the avirulent line. Consistent with our hypothesis, drug treatment was disproportionately effective against the less virulent parasites. Treatment did not affect the relative transmission advantage of the virulent line. Neither of the lines contained known mutations conferring classical drug resistance.

**Conclusion:**

Drug-sensitivity of malaria parasites can be virulence-dependent, with virulent parasites more likely to survive sub-optimal treatment. If this proves to be general for a variety of drugs and parasite species, selection imposed by sub-optimal drug treatment could result in the evolution of more aggressive malaria parasites.

## Background

Patients in endemic areas often carry malaria parasites while having levels of antimalarial drugs in their blood that fail to eliminate all parasites. This can be due to the presence of residual levels of drugs when a new infection is acquired [1,2], because recommended treatment could not clear all parasites [3-6] or because of inadequate treatment (for example from low quality drugs [7,8] or poor compliance). Due to variable metabolic drug uptake [9,10], recommended doses may not be adequate for all sub-groups, such as children or pregnant women [4,11,12]. Even during supervised treatment with potent and high quality drugs, including artemisinin-based combination therapies, patients can be cured from disease symptoms but low numbers of parasites to survive and transmit [5,13,14]. In all these cases, drugs are not maintained at sufficient doses for long enough to kill all parasites.

**Figure 1:**
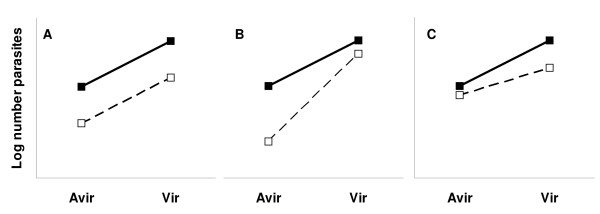
**Possible outcomes for the relation between drug sensitivity and virulence**. Without drug treatment (black symbols), infections with the virulent parasite line (Vir) produce more parasites compared to the avirulent line (Avir). If both lines are affected similarly by drug treatment (open symbols), the proportionate difference between them will be unaffected by drug treatment (1A). If the virulent line is less sensitive to drug treatment, the reduction in parasite numbers will be disproportionately greater for the avirulent line (1B). If the virulent line is more sensitive to drug treatment, the reduction in parasite numbers will be disproportionately greater for the virulent line (1C).

Exposure of parasites to sub-optimal drug levels can facilitate the evolution of classical drug resistance, where resistant parasites survive drug treatment through well-studied mechanisms, such as target-site mutations and detoxification or efflux pathways [15]. In what follows, such mechanisms are referred to as 'classical drug resistance'. However, genetically-encoded parasite traits other than classical resistance could also influence survival in drug-treated infections [16-20] and, hence, be subject to drug-induced selection pressures. For instance, malaria parasites vary in virulence (the harm caused to hosts as a consequence of infection) [21-23], the molecular basis of which is beginning to be understood [24-26]. Here it is proposed that virulence affects drug sensitivity. For instance, in some cases, parasite lines which replicated rapidly or which can persist at higher densities for longer are associated with more severe disease (virulence) [21,27,28]. This could make them less vulnerable to drug treatment if they can rapidly recover high parasite densities after a bout of drug-induced mortality. Alternatively, rapid replication could render parasites more vulnerable to metabolic disruption and thus, greater drug-induced mortality. In either case, the evolution of higher or lower virulence could be a consequence of drug-induced selection. Testing whether a given drug regime has differential effects on virulent and avirulent parasites is the first key step in testing the hypothesis that chemotherapy has the potential to impose selection for parasite traits other than those involved in classical resistance (Figure 1). Such selection processes may be occurring in the field but are difficult to study due to various confounding factors, including the presence of multiple, interbreeding parasite genotypes. Therefore, *in vivo *experiments using an animal model were designed to test if sensitivity to drug treatment can be virulence-dependent.

## Methods

### Terminology

#### Drug resistance

The WHO defines drug resistance as "the ability of a parasite strain to survive or multiply in the presence of drug concentrations that normally destroy parasites of the same species or prevent their multiplication, provided that the drug was properly absorbed and parasites exposed to it" [29]. This definition includes all parasite traits that affect the survival or transmission of parasites exposed to drugs. Drug resistance in malaria is acquired through selection of mutants that are favoured under conditions of drug exposure. This includes mutations directly related to the action of the drug ('classical drug resistance'), but also other parasite traits that, directly or indirectly, may confer a survival and transmission advantage in the presence of drugs (non-classical drug resistance). As was hypothesised above, parasite replication rate could be an example of such a non-classical resistance trait, whereas target site mutations, and efflux and detoxification mechanisms are examples of classical resistance. Non-classical drug resistance is the subject of this study.

#### Sub-curative vs. sub-optimal

Ideally, drug treatment achieves two things: restoration of patient health and clearance of parasites. In the literature, the term 'cure' is used variably to refer to either or both of these outcomes. Therefore, the term 'sub-curative drug-treatment' is ambiguous. For clarity, the term 'sub-optimal drug-treatment' is used here and specifically defined as drug treatment that allows classically drug-sensitive parasites to survive, with or without eliminating disease symptoms.

### Experimental overview and rationale

Here, the first experimental tests of how genetically related parasites, differing in virulence, perform under a range of drug treatment regimes are presented. It is well known that serial passage increases parasite virulence [30,31]. This makes it possible to derive parasites, which differ in virulence but come from the same clonal lineage. Thus, the effect of chemotherapy on asexual- and transmission-stage densities can be tested on genetically similar parasites differing in virulence. To mimic the possible causes of sub-optimal treatment that can occur in the field (reviewed in the Introduction), parasites were challenged with sub-optimal doses and sub-optimal duration of drug treatment when symptoms first appeared.

### Parasites

The original *Plasmodium chabaudi *isolate was obtained from a thicket rat from the Central African Republic [32]. Genotype CW_avir _(CW175) was obtained after cloning of the wild isolate and 4 passages in mice. CW_vir _(CW202) was derived from CW_avir _by 11 serial passages in mice [30] and became more virulent in the process [33]. Samples of CW_avir _and CW_vir _were passaged a further three times in C57Bl/6J mice before the experiments described here. None of the parasite lines involved, nor any of their ancestors had been exposed to antimalarial drugs prior to these experiments.

### Experimental set-up

Drug sensitivity to the antimalarial drug pyrimethamine was tested *in vivo *using 6–10 week old C57Bl/6J female mice (Harlan), with access to food (41B maintenance diet, Harlan) and drinking water supplemented with 0.05% para-amino benzoic acid [34]*ad libitum*. Mice were infected with 10^5 ^parasites of either CW_vir _or CW_avir _by intraperitoneal injection and given treatment with the antimalarial drug pyrimethamine from day 5 after infection, when first symptoms of disease occurred. The experimental setup is shown in Table 1. Pyrimethamine was administered during four consecutive days in experiment 1 (five mice per group) and experiment 2 (four mice per group) at doses of 0, 1, 3 and 5 mg/kg or, in experiment 3, on a single day at doses of 0, 4, 8, 12 or 20 mg/kg (four mice per group). In experiment 1, four mice infected with CW_vir _were excluded from analysis, three of these were severely anaemic and had to be euthanized early in the experiment (dose 0, n = 1; dose 1, n = 2) and parasites were not detected at time of treatment in another mouse (dose 5; Table 1). In experiment 2, all four mice in the untreated CW_vir _group had to be euthanized a few days after the peak of infection on day 11 (n = 1) or 12 (n = 3), and available data have been used in the analysis unless indicated otherwise. Pyrimethamine was dissolved in dimethyl sulphoxide at the required concentration and injected intraperitoneally with a maximum total volume of 50 μL. To monitor treatment efficacy, parasite dynamics and virulence (weight loss, anaemia) were followed until three weeks post infection, when mice no longer show disease symptoms. Asexual parasites were counted per 1000 RBCs by examination of Giemsa-stained thin smears from tail bleeds. Red blood cell density was determined by flow cytometry (Beckman Coulter) and used to calculate parasite density/mL of blood. Blood samples for RNA (10 μL) and DNA (5 μL) extraction were stored to quantify gametocytes by quantitative reverse-transcriptase PCR and for sequencing to detect pyrimethamine resistance mutations.

**Table 1:** Treatment groups and sample sizes for experiments 1, 2 and 3

		**Nr mice exp 1**		**Nr mice exp 2**	
**Days**	**Dose**	**CW**_avir_	**CW**_vir_	**CW**_avir_	**CW**_vir_
4	0	5	5*	4	4#
4	1	5	5**	4	4
4	3	5	5	4	4
4	5	5	5§	4	4
		**Nr mice exp 3**			
**Days**	**Dose**	**CW**_avir_	**CW**_vir_		

1	0	4	4		
1	4	4	4		
1	8	4	4		
1	12	4	4		
1	20	4	4		

Days: duration of pyrimethamine treatment in days, starting from day 5 after infection. Dose: mg/kg/day. Nr mice: sample sizes for each group, with sample sizes for each experiment. * Each symbol indicates a mouse excluded from analysis, euthanized early in the experiment because of severe anaemia. ^§ ^1 mouse excluded, no parasites detected at time of treatment. ^# ^4 mice euthanized on d11 and d12 after infection, data have been used where possible.

### Nucleic acid extraction and PCR analysis of gametocytes

Ten μL blood samples were added to 30 μL nucleic acid purification lysis solution (Applied Biosystems) and 15 μL PBS, gently mixed and stored at -80°C. RNA was extracted using the ABI Prism 6100^® ^[35] and cDNA was obtained by reverse transcriptase PCR (high capacity cDNA archive kit, Applied Biosystems) according to manufacturer's protocols. Gametocytes were counted using quantitative real-time PCR with primers based on the gametocyte-specifically expressed gene PC302249.00.0 [36].

**Figure 2:**
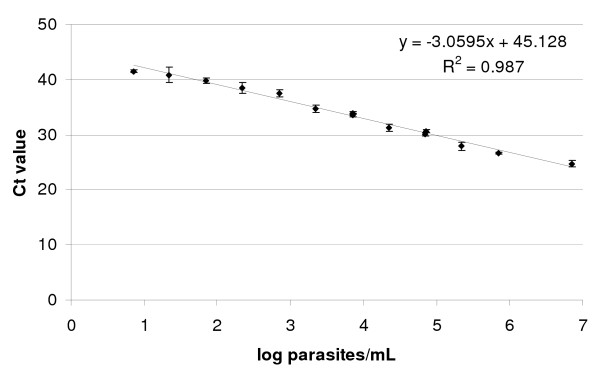
**PCR quantification of *P. chabaudi *gametocytes**. Average threshold value (Ct) ± standard deviation for *P. chabaudi *gametocyte control samples tested in 25 different 96-well plates for quantitative PCR.

During qPCR analysis for gametocyte quantification, three different dilution series of positive control samples, initiated from different mice with *P. chabaudi *gametocytes, were tested in concentrations ranging from 7 to 7*10^6 ^gametocytes/mL. The average threshold cycle (Ct) values ± 1 standard deviation are shown in Figure 2. Gametocyte numbers have a high correlation to Ct values (R^2 ^= 0.99; p < 0.0001). The absolute detection limit for the assay was determined to be at least 700 gametocytes/mL of blood. Lower gametocyte densities were detected in 88% (221 gametocytes/mL), 67% (71/mL), 43% (22/mL) and 12% (7/mL) of the tested control samples. This decline in probability of detection is most likely related to the probability of target nucleic acid being present in the 10 μL blood sample.

### Classical drug resistance genotyping

Pyrimethamine is an antifolate drug that inhibits the enzyme dihydrofolate reductase (DHFR) that is essential in the folic acid pathway, ultimately leading to pyrimidine synthesis. Pyrimethamine inhibits parasite multiplication but does not directly kill gametocytes. As with other malaria parasite species, resistance to pyrimethamine in *P. chabaudi *is conferred by point mutations in the *dhfr *gene, resulting in conformational changes of the enzyme and decreased binding capacity of the drug [37,38]. Samples from the day of infection and day 21 were analysed to detect resistance mutations in the *dhfr *gene, using the following protocol. DNA was extracted using InstaGene Matrix (BioRad) according to the standard protocol for DNA preparation from whole blood. The extracted DNA, forward primer pcdhfr-10 5'-GCTATTTCTTTCTACATTTGC-3' and reverse primer pcdhfr-11 5'-TTTAAAATGATGAGCATGCTC-3' were used to amplify the region of the *dhfr *gene that contains possible mutations involved in pyrimethamine resistance. The PCR reaction included 13.75 μL water, 10 μL 5× PCR buffer (Promega), 3 μL 25 mM MgCl_2_, 1 μL dNTPs 10 mM, 0.25 μL Taq 5 u/μL, 1 μL of each primer 10 μM and 20 μL DNA sample. The temperature programme used for amplification was 95°C for 60 seconds, then 30 cycles of each 95°C for 60 seconds, 52°C for 60 seconds, 65°C for 60 seconds and ending with 65°C for 600 seconds and storage at 4°C. PCR products were purified using Qiaquick PCR purification kit (Qiagen Ltd. UK) according to manufacturer's protocol and sequenced.

### Statistics

The maximum and cumulative measures of parasite and gametocyte density achieved by each infection during the 21-day experiments were used as response variables in the analysis. *Plasmodium chabaudi *has a cell-cycle of approximately one day for asexual parasites, therefore, cumulative parasite density is a measure of the total number of parasites present during a defined period of the infection. Gametocyte density correlates positively with infectivity to mosquitoes [21,39] and, when summed over the course of an infection, provides a measure of transmission potential. Cumulative parasite and gametocyte counts were log_10_-transformed to meet assumptions of normality and homogeneity of variance. Analyses were performed using general linear models in SPSS version 14.0 (SPSS Inc., Chicago, USA). Full models for analysis of parasite data included the main effects of clone (CW_avir _or CW_vir_; virulence classification) and dose, their 2-way interaction, experiment (1–3) as a random factor, and the covariates weight and anaemia at the day of infection as well as parasite density at the start of treatment. The clone × dose interaction indicates that CW_vir _and CW_avir _were differentially affected by drug dose, the hypothesis under test (Figure 1). Models were minimized using step-wise deletion.

## Results

### Virulence of ancestral and derived parasites in the absence of drug-treatment

**Figure 3:**
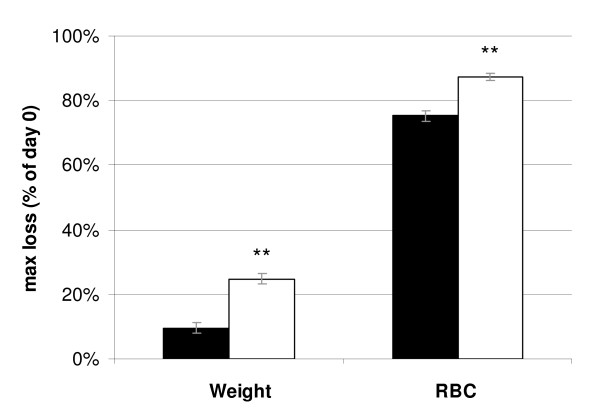
**Infections with CW_vir _resulted in greater weight loss and red blood cell loss than CW_avir_**. Virulence measures for *P. chabaudi *CW_avir _and CW_vir _without drug treatment. Mean ± s.e. values over 13 (CW_avir_) and 12 (CW_vir_) infections combined for experiments 1, 2 and 3 are shown. Statistical difference between lines after adjustment for between-experiment differences (experiment) are at 0.001 level (**).

As found previously [33], CW_vir _was indeed more virulent in the absence of chemotherapy, generating greater weight loss and anaemia than CW_avir _(Figure 3; mean difference ± s.e. of maximum weight loss: 15 ± 2% of starting body mass; F_1,21 _= 68.6, p < 0.001; mean difference ± s.e. 12 ± 2% of starting RBC density; F_1,21 _= 47.9, p < 0.001).

### Asexual parasites

**Figure 4:**
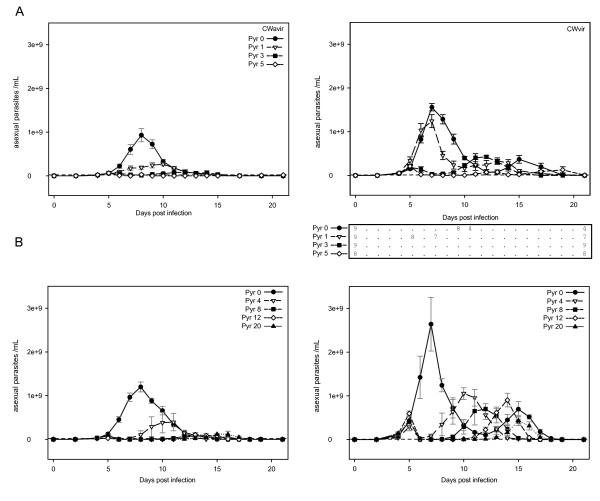
**Effects of pyrimethamine treatment on parasite dynamics of virulent and avirulent *P. chabaudi***. Number of asexual parasites/ml of blood over time ± s.e. for increasing pyrimethamine doses. PYR0 = no drug, PYR1, 3, 4, 5, 8, 12 and 20 indicate daily doses of pyrimethamine in mg/kg. 4A. 4-day pyrimethamine treatment, combined for experiments 1 and 2 with 9 mice in all treatment groups, except CW_vir _dose 5 (n = 8). Death of mice during the experiment is indicated by the table below the CW_vir _graph. 4B. Experiment 3, 1-day treatment with 4 mice in each treatment group.

**Figure 5:**
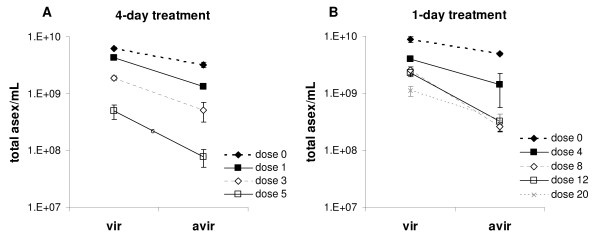
**Effects of pyrimethamine treatment on total asexual parasite density during an infection**. Total asexual parasites/mL blood summed for days 6–21 after treatment ± standard error. PYR0 = no drug, PYR1, 3, 4, 5, 8, 12 and 20 indicate daily doses of pyrimethamine in mg/kg. 5A: experiments 1 and 2 combined. 5B: Experiment 3.

In the absence of drugs, CW_vir _achieved higher maximum asexual parasite densities than CW_avir _(mean difference ± s.e. 9.9 ± 2.5 × 10^8^, F_1,21 _= 21.5, p < 0.001). Drug treatment reduced maximum asexual parasite density and delayed peak parasite density in a dose-dependent manner (Figure 4). At all doses, cumulative asexual parasite density was reduced by drug treatment (dose effect, F_7,90 _= 58.9, p < 0.0001), but remained significantly higher in CW_vir _than CW_avir _infections (Figure 5; clone effect, F_1,90 _= 153.4, p < 0.0001). The relative reduction in parasites densities induced by the different doses differed between the CW_vir _and CW_avir _(clone × dose interaction F_7,90 _= 3.6, p = 0.002), with the impact disproportionately greater on CW_avir_. Thus, sub-optimal chemotherapy enhanced the relative difference in cumulative parasite densities between the two clonal lineages (Figure 5).

### Gametocytes

**Figure 6:**
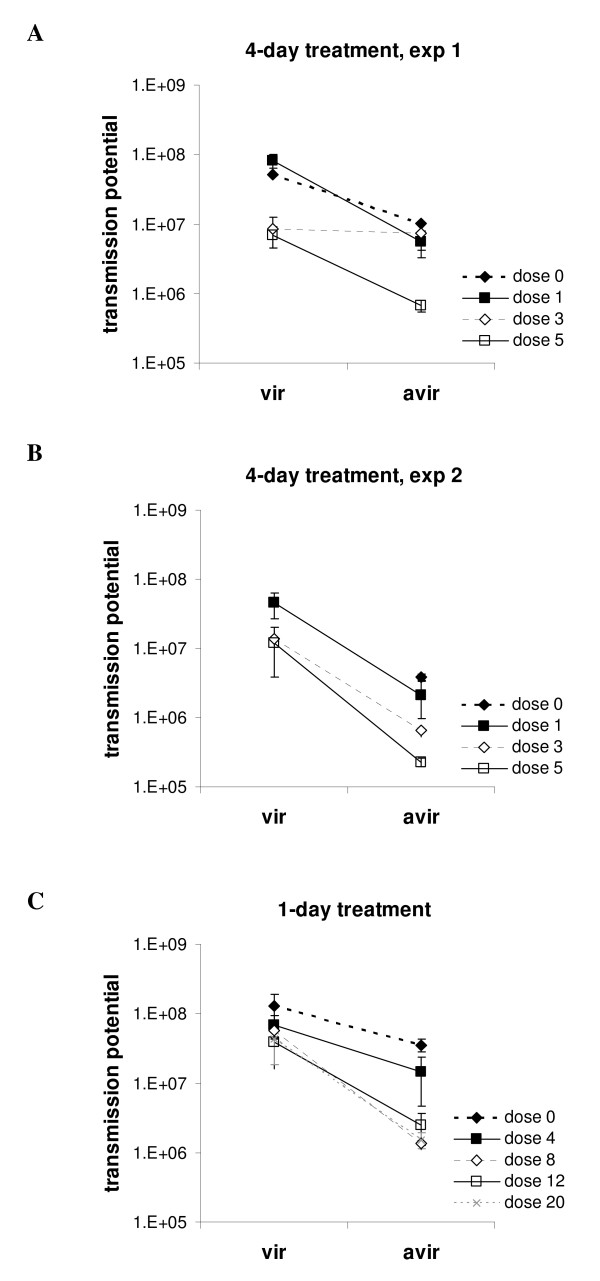
**Effects of pyrimethamine treatment on total gametocyte density during an infection**. Total gametocytes/mL blood ± s.e. summed over the duration of infection. PYR0 = no drug, PYR1, 3, 4, 5, 8, 12 and 20 indicate daily doses of pyrimethamine in mg/kg. Figure 6A and B. 4-day treatment experiments 1 and 2, summed for days 6–21. Data for the untreated CW_vir _group is missing as a result of host death on days 11 and 12. Figure 6C. Experiment 3, 1-day treatment, summed for days 6–19.

In the absence of drug treatment, CW_vir _infections had higher peak gametocyte densities (mean difference ± s.e. 34.4 ± 12.8 × 10^6^, F_1,19 _= 7.3, p = 0.014) as well as higher total gametocyte production (Figure 6; F_1,15 _= 30.9, p < 0.001) than CW_avir _infections.

Total gametocyte numbers were significantly reduced by treatment (dose effect, F_7,82 _= 16.0, p < 0.001) and the higher transmission potential for CW_vir _is generally maintained under conditions of drug treatment (clone effect F_1,82 _= 51.7, p < 0.001).

Overall the experiments, the effect of drug treatment on the two parasite lines was marginally different (clone × dose interaction F_7,82 _= 2.1, p = 0.06). However, breaking these data down by experiment showed that there was no general pattern (Figure 6). Gametocyte densities in the third experiment, which involved one day of treatment show similar patterns (Figure 6C) to those seen in the asexual densities (Figure 5), but the disproportionate reductions in transmission potential of CW_avir _at the higher doses was not statistically significant (clone × dose F_4,17 _= 1.62, p = 0.196). Due to loss of data for the untreated CW_vir _group in experiment 2 (see Methods), it is not possible to determine the differences in transmission potential between the two lines in the absence of treatment (Figure 6B). Among the remaining (treated) groups, the different doses had similar effects on both CW_vir _and CW_avir _(clone × dose F_2,20 _= 0.11, p = 0.901). In experiment 1, the clone differences did vary with treatment (clone × dose F_3,24 _= 9.45, p < 0.001), but inspection of Figure 6A shows that chemotherapy is causing similar proportionate reductions in gametocyte numbers for both lines except at an intermediate dose, which was disproportionately effective against CW_vir _(or ineffective against CW_avir_). The cause for this effect is unknown and it was not observed in the repeat experiment (Figure 6B).

### *Dhfr *mutations

Mutations known to confer resistance to the drug pyrimethamine in *P. chabaudi *[37,38] in parasites of either clonal lineages were not observed, either at the start of the infections, or among parasites surviving treatment and present on day 21 post-infection, the last day of the experiment.

## Discussion

In this study, the drug sensitivity of two parasite lines, one derived from the other by serial passage [30] were compared. The virulent derived line was significantly less sensitive to sub-optimal pyrimethamine treatment (Figures 4 and 5). This decreased sensitivity to pyrimethamine was not associated with the presence at detectable levels of mutations known to confer classical drug resistance [37,40]. Because of this, and because these parasite lines had never previously been exposed to pyrimethamine and because possible between-host variation in immune response and bioavailability have been controlled for by using inbred hosts, these data provide evidence for 'non-classical' drug resistance in malaria parasites.

These data raise several potentially important questions. First, is the differential susceptibility to drugs due to parasite factors associated with virulence? Higher parasite densities at the time of treatment could be associated with both virulence and the ability to recover from sub-optimal chemotherapy [17-20]. However, parasite density at time of treatment was included as a covariate in all analyses and it is therefore unlikely that higher parasite densities *per se *are responsible for the patterns reported. Clearly, with a comparison of two lines, it is not possible to attribute the cause of the differential susceptibility to any specific difference between CW_vir _and CW_avir_. Nonetheless, these two lines have almost identical genotypes and the most obvious difference between them is that the derived line is more virulent. Establishing whether virulence is responsible for the 'non-classical drug resistance' observed requires more data.

Second, could sub-optimal chemotherapy impose selection in favour of more virulent parasites? Subject to the normal cautions about generalizing from animal models (reviewed in this context by Wargo *et al *[41]), these data suggest it could. The avirulent parasites were disproportionately suppressed (Figure 5), implying that within-host selection imposed by drug pressure will favour virulent parasites. To determine directly whether this within-host selective advantage translates into a between-host (transmission) advantage requires assays involving mosquitoes, a high priority for future work. We found no consistent evidence that the transmission stage densities of the virulent parasite line were less affected by treatment than those of the avirulent line, but the relationship between gametocyte density and mosquito infection can be influenced by several non-mutually exclusive factors (e.g. [42-45]), and malaria parasites can compensate for mild levels of drug-induced mortality by the production of more transmission stages [46-48]. We expect that if co-infection of both the virulent and avirulent line were drug treated, the within-host selective advantage of the virulent line would translate into greater relative transmission.

Chemotherapy could also drive virulence evolution by a completely different evolutionary mechanism. Virulence theory assumes that whilst virulence and transmission are positively correlated in the absence of host death, excessively virulent parasites risk truncating their infectious period by prematurely killing their host. Under this scenario, it is host death that restrains the evolution of ever more virulent parasites. A logical consequence of this view is that interventions which keep hosts alive reduce this restraining force, while still allowing parasites to benefit from the transmission rewards of increasing their virulence. Thus life-protecting interventions have the potential to allow more virulent strains to circulate in malaria populations [49]. This hypothesis is supported by observations that virulence increases with serial passage [30,31], and theoretical analysis of the evolutionary consequences of vaccination [50-52] or chemotherapy [53,54] predict virulence to increase. If sub-optimal drug treatment reduces the costs of virulence (hypothesized) as well as conferring a survival advantage on virulent parasites (as the data reported here indicate), chemotherapy could be a potent force for the evolution of more virulent parasites.

These ideas and the data reported here, raise several questions that could be explored theoretically. First, will there be positive feedback, whereby the frequency of sub-optimally treated infections will rise as the virulent parasites favoured by sub-optimal therapy become more common? Can this process be halted by increasing drug doses? Second, combination therapy is now recommended by the WHO because it reduces the probability that classical drug-resistance mutations can emerge in the first place. By preventing classical resistance from arising, is combination therapy making virulence evolution more likely? There will almost certainly already be standing genetic variation in virulence in nature on which selection can act. Third, will classical resistance, when it does arise, be more likely to appear in more virulent parasites? Fourth, could virulence-related susceptibility to drugs act as a form of cross-resistance which continue to protect parasites when policy switches to new drugs after failure of current combinations? The preliminary data reported here, are obviously far from the final word, but given the potential importance of the problem, these preliminary data strongly argue for further investigation of questions like these, and of course further empirical investigation of the phenomenon of virulence-mediated drug sensitivity.

## Conclusion

Virulent parasites were disproportionately less susceptible to sub-optimal drug treatment than were less virulent parasites. It is important to determine whether virulence-dependent drug sensitivity occurs across a variety of drugs, treatment regimes and parasite species. If it does, selection imposed by sub-optimal drug treatment could cause the evolution of more virulent parasites, rendering future infections more aggressive.

## Competing interests

The authors declare that they have no competing interests.

## Authors' contributions

PS designed the study, collected, analysed and interpreted the data and drafted the manuscript. BC assisted in data collection. SR and AR assisted in conception of the study, interpretation of data and drafting the manuscript. All authors read and approved the final manuscript.
